# *Staphylococcus aureus *sigma B-dependent emergence of small-colony variants and biofilm production following exposure to *Pseudomonas aeruginosa *4-hydroxy-2-heptylquinoline-*N-*oxide

**DOI:** 10.1186/1471-2180-10-33

**Published:** 2010-01-30

**Authors:** Gabriel Mitchell, David Lalonde Séguin, Ann-Elise Asselin, Eric Déziel, André M Cantin, Eric H Frost, Sophie Michaud, François Malouin

**Affiliations:** 1 Centre d'Étude et de Valorisation de la Diversité Microbienne (CEVDM), Département de biologie, Faculté des sciences, Université de Sherbrooke, Sherbrooke, QC, J1K 2R1, Canada; 2 INRS-Institut Armand Frappier, Laval, QC, H7V 1B7, Canada; 3 Unité de Recherche Pulmonaire, Faculté de médecine et des sciences de la santé, Université de Sherbrooke, Sherbrooke, QC, J1H 5N4, Canada; 4 Département de Microbiologie et d'Infectiologie, Faculté de médecine et des sciences de la santé, Université de Sherbrooke, Sherbrooke, QC, J1H 5N4, Canada

## Abstract

**Background:**

*Staphylococcus aureus *and *Pseudomonas aeruginosa *are often found together in the airways of cystic fibrosis (CF) patients. It was previously shown that the *P. aeruginosa *exoproduct 4-hydroxy-2-heptylquinoline-*N-*oxide (HQNO) suppresses the growth of *S. aureus *and provokes the emergence of small-colony variants (SCVs). The presence of *S. aureus *SCVs as well as biofilms have both been associated with chronic infections in CF.

**Results:**

We demonstrated that HQNO stimulates *S. aureus *to form a biofilm in association with the formation of SCVs. The emergence of SCVs and biofilm production under HQNO exposure was shown to be dependent on the activity of the stress- and colonization-related alternative sigma factor B (SigB). Analysis of gene expression revealed that exposure of a prototypical *S. aureus *strain to HQNO activates SigB, which was leading to an increase in the expression of the fibronectin-binding protein A and the biofilm-associated *sarA *genes. Conversely, the quorum sensing accessory gene regulator (*agr*) system and the α-hemolysin gene were repressed by HQNO. Experiments using culture supernatants from *P. aeruginosa *PAO1 and a double chamber co-culture model confirmed that *P. aeruginosa *stimulates biofilm formation and activates SigB in a *S. aureus *strain isolated from a CF patient. Furthermore, the supernatant from *P. aeruginosa *mutants unable to produce HQNO induced the production of biofilms by *S. aureus *to a lesser extent than the wild-type strain only in a *S. aureus *SigB-functional background.

**Conclusions:**

These results suggest that *S. aureus *responds to HQNO from *P. aeruginosa *by forming SCVs and biofilms through SigB activation, a phenomenon that may contribute to the establishment of chronic infections in CF patients.

## Background

Although cystic fibrosis (CF) is fundamentally a genetic disorder, the majority of patients with CF may ultimately succumb to respiratory failure subsequent to chronic bacterial infections [1]. In early childhood, lungs of CF patients are often infected with *Staphylococcus aureus *and *Haemophilus influenzae*, but these organisms are usually outnumbered by *Pseudomonas aeruginosa *as patients become older. However, *S. aureus *often persists in the airways of CF patients and the role of *S. aureus *in the progression of CF patients to respiratory failure is not yet understood whereas infections with *P. aeruginosa *is considered as one of the main factors for a decline in lung function and mortality [1]. Interestingly, both organisms are commonly co-isolated from CF airways [2,3].

Infections with mixed microbial communities are common, although very little is known about the importance and the impact of interspecies interactions [4]. It is now becoming obvious that the different bacteria found in CF airways interact together in several different ways [5-10]. One possibility is that polymicrobial interactions influence pathogenic processes such as biofilm formation [1,9]. Accordingly, the biofilm lifestyle is now recognized as an integrated and complex polymicrobial community and it is thought that cell-to-cell interspecies signals play a role in the control of this behavior [11].

It has recently been shown that prolonged growth of *S. aureus *with physiological concentrations of the *P. aeruginosa *exoproduct 4-hydroxy-2-heptylquinoline-*N*-oxide (HQNO) selects for a sub-population of slow-growing respiratory deficient *S. aureus *named small-colony variants (SCVs) [2]. The respiratory deficiency of SCVs provides resistance to aminoglycoside antibiotics, which can contribute to microbial persistence during antibiotherapy [12]. Furthermore, it has been recently demonstrated that SCV selection is a survival strategy of *S. aureus *against *P. aeruginosa *[13]. *S. aureus *SCVs are often isolated from chronic infections [12], as in the case of lung infections of CF patients [14-16]. Several studies have shown that *S. aureus *SCVs possess an increased capacity to invade and persist in host cells [14,15,17], which is thought to confer the bacterium protection against the immune system and the action of antibiotics [17,18]. Using SCVs isolated from cystic fibrosis patients, we have previously demonstrated that the alternative transcription sigma factor B (SigB) influences the expression of several virulence factors and is associated with an increased ability to adhere, invade and persist within host cells [15,19]. Furthermore, our more recent results suggest that SigB is involved in the emergence of SCVs under aminoglycoside pressure [20], which suggests that the appearance of SCVs may be a regulated process influenced by environmental cues. Our current hypothesis is that SigB plays an important role in the establishment of chronic and difficult-to-treat *S. aureus *infections.

SigB is involved in the response to environmental stresses such as during stationary phase, heat exposure and change in osmotic pressure [21]. Moreover, the activity of SigB positively influences the expression of several cell-surface proteins whereas it down-regulates a variety of toxins [22], which suggest an important role for SigB in pathogenesis. The effect of SigB on virulence gene expression can be direct or indirect, since the genes regulated by SigB also include at least another global regulator of virulence, *sarA *(Staphylococcal accessory regulator) [22,23]. SarA modulates the expression of several virulence factors either by stimulating RNAIII transcription or by pathway(s) independent of the *agr *(accessory gene regulator) system [24]. In turn, it is proposed that the quorum-sensing *agr *system controls the transition from colonization to dissemination by up-regulating the expression of several exotoxins and proteolytic enzymes and by repressing the expression of cell-surface proteins involved in colonization [25]. *agr *[26], SigB [27,28] and SarA [29] are known to influence the formation of biofilms by *S. aureus*.

At least two different mechanisms of biofilm formation exist in *S. aureus *[26,29-33]. The first mechanism implies the production of the polysaccharide intercellular adhesin (PIA), which requires the *ica *gene cluster, whereas the second mechanism is *ica*-independent. With opposite effects, SarA and *agr *are both involved in the *ica*-independent mechanism of biofilm formation. SarA is thought to be indirectly required for the initial attachment step to biological matrices [29,32,33], while *agr *is controlling the dispersal process of biofilms [26]. Recently, Lauderdale *et al. *[30] have shown that SigB is an essential regulator of the *ica*-independent biofilm formation and suggested that SigB acts upstream of the *agr *system, allowing the formation of biofilm to be regulated as a function of environmental factors. Noteworthy, biofilms have been linked to chronic infections, especially in the case of those found in the airways of CF patients [1,34], and an increased formation of biofilms has been associated with the SCV phenotype [20,35].

The aim of this study was to investigate the association between the activity of SigB, the emergence of SCVs and biofilm production in *S. aureus *when exposed to *P. aeruginosa *HQNO.

## Results

### HQNO inhibits the growth of normal strains and provokes the emergence of SCVs in *S. aureus*

**Figure 1:**
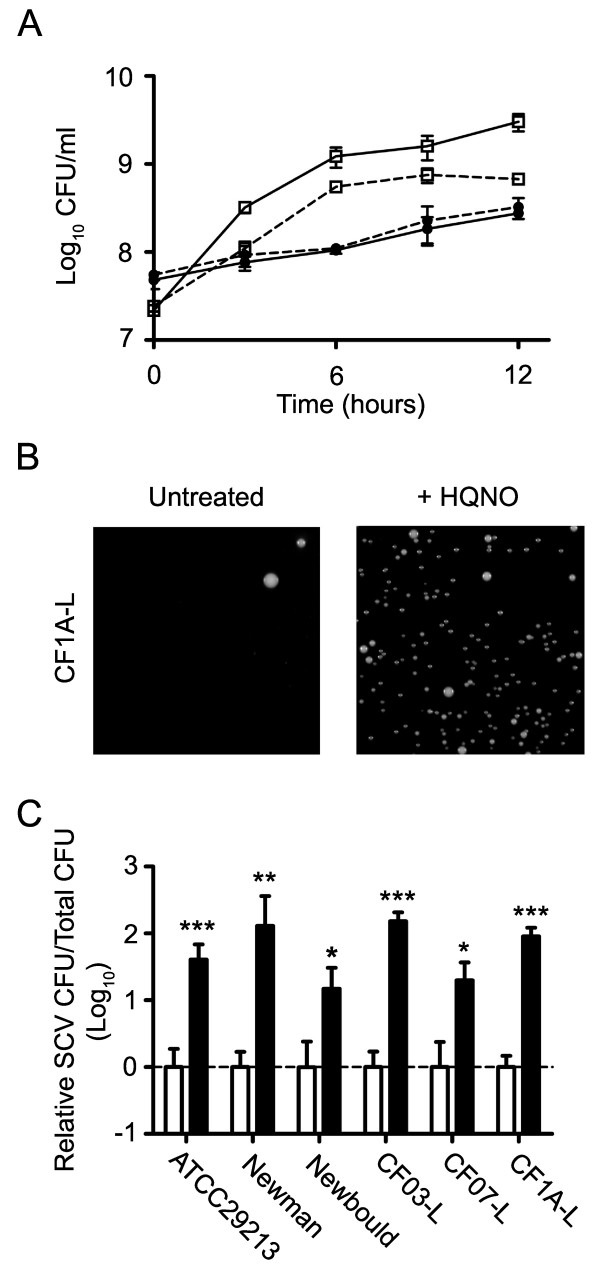
**HQNO inhibits the growth of normal *S. aureus *strains and provokes the emergence of SCVs**. (A) Growth curves of the normal strain CF1A-L (□) and the SCV CF1D-S (●) exposed (dotted lines) or not (solid lines) to 10 μg/ml of HQNO. (B) Pictures show SCV colonies grown on agar containing a selective concentration of gentamicin following or not an overnight treatment of strain CF1A-L with 10 μg/ml of HQNO. (C) Relative number of SCV CFUs recovered after 18 h of growth from strains ATCC 29213, Newman, Newbould, CF03-L, CF07-L and CF1A-L following (black bars) or not (open bars) treatments with 10 μg/ml of HQNO. Data are presented as means with standard deviations from at least three independent experiments. Results are normalized to the non exposed condition for each strain (dotted line). Significant differences between untreated and HQNO-treated conditions are shown (*, *P *< 0.05; **, *P *< 0.01; ***, *P *< 0.001; unpaired *t-*test).

Fig. 1 confirms that HQNO suppresses the growth of *S. aureus *and causes the emergence of SCVs. Isolates CF1A-L and CF1D-S are two related strains co-isolated from a CF patient which have a normal and a SCV phenotype, respectively (see Methods). At a concentration of 10 μg/ml, HQNO significantly attenuated the growth of CF1A-L (*P *< 0.01 from 6 to 12 h of growth; two-way ANOVA followed by a Bonferroni's post test) whereas HQNO had no apparent effect on the growth of CF1D-S which was already significantly slower than that of CF1A-L in the absence of HQNO (*P *< 0.001 from 6 to 12 h of growth; two-way ANOVA followed by a Bonferroni's post test) (Fig. 1A). Similar observations were also reproduced with other strains (two normal and one SCV; data not shown). Fig. 1B shows that an overnight treatment with HQNO provokes the emergence of SCVs from CF1A-L, as determined by plating the culture on solid medium containing a concentration of gentamicin selective for the SCV phenotype. Very little or no SCV were detected on gentamicin plates when cultures were not exposed to HQNO (Fig. 1B). Hence, this technique allowed detection and quantification of SCVs emerging during the growth of normal bacteria exposed or not to HQNO. This approach was thus used to distinguish the transitory suppression of growth of normal *S. aureus *by HQNO from the emerging slow-growing SCVs for which gentamicin resistance and slow growth persist even after removal of HQNO. Fig. 1C shows that 10 μg of HQNO/ml significantly increased the presence of SCVs in cultures of the prototypical strains ATCC 29213, Newman and Newbould as well as of the other normal strains isolated from CF patients CF03-L, CF07-L and CF1A-L. Differences in HQNO-mediated SCV emergence between strains were not significant, except between ATCC 29213 and Newbould (*P *< 0.01; one-way ANOVA followed by a Tuckey's post test). These results corroborate that HQNO generally suppresses the growth of normal *S. aureus *populations and provokes the emergence of SCVs from strains of different origins.

### HQNO stimulates biofilm production in normal strains but does not alter high biofilm production in SCVs

**Figure 2:**
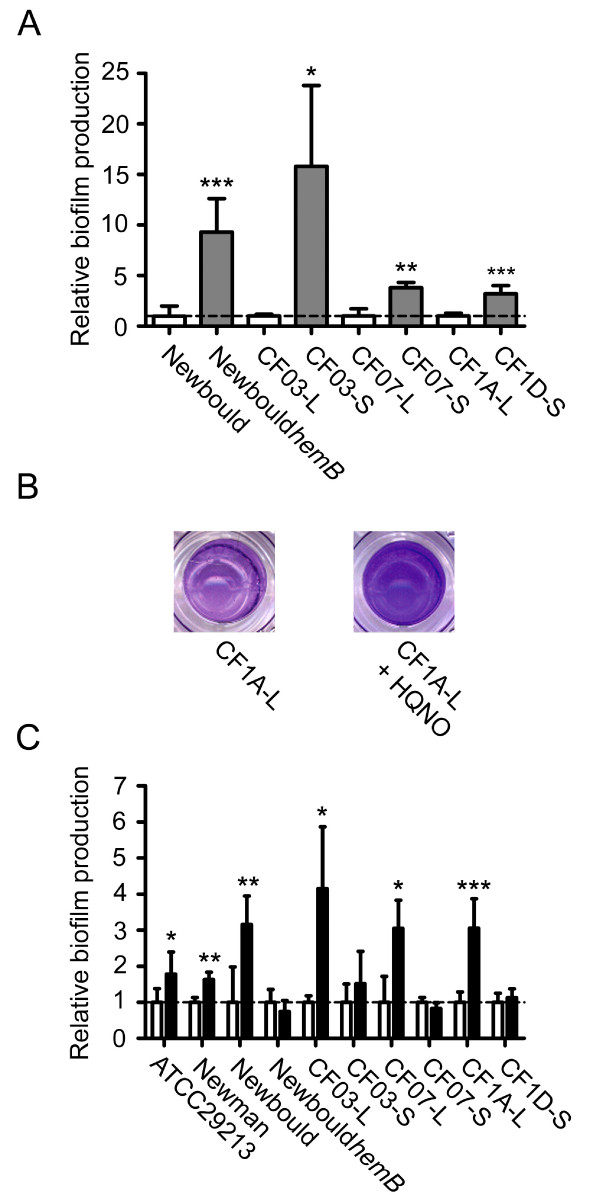
**HQNO stimulates biofilm production in normal strains but does not alter high biofilm production in SCVs**. (A) Relative biofilm production in related normal (open bars) and SCV (grey bars) strains. Results are normalized to the normal strain for each pair (dotted line). (B) Pictures show the biofilm formation of the normal strain CF1A-L in the absence or in the presence of HQNO as detected by crystal violet staining. (C) Relative biofilm production in strains exposed (black bars) or not (open bars) to 10 μg/ml of HQNO. Results are normalized to the unexposed condition for each strain (dotted line). Data are presented as means with standard deviations from at least three independent experiments. Significant differences between normal and SCV strains (-L and -S suffixes, respectively) or between unexposed and HQNO-exposed conditions are shown (*, *P *< 0.05; **, *P *< 0.01; ***, *P *< 0.001; unpaired *t-*test).

Several pairs of related normal and SCVs strains were used in order to study the effect of HQNO on biofilm production by *S. aureus*. Fig. 2A shows that SCVs produce significantly more biofilm than their normal counterparts. The use of the strain Newbould*hemB *(which is a stable laboratory-derived SCV) ensured that SCVs (and not revertants) are indeed responsible for this increase in biofilm production (at least in the case of Newbould*hemB*). Furthermore, as shown in Mitchell *et al. *[20], supplementation of the SCV strains CF03 and CF07 with menadione abolished this phenomenon and thus demonstrated that if there was a reversion of SCVs to the normal phenotype, the biofilm production would be greatly reduced.

Besides, the presence of HQNO at 10 μg/ml did stimulate biofilm production in the normal strains (Fig. 2B-C). This observation was statistically significant for the normal strains ATCC 29213, Newman, Newbould, CF03-L, CF07-L and CF1A-L whereas HQNO had no detectable effect on the already high biofilm production of the SCV strains Newbould*hemB*, CF03-S, CF07-S and CF1D-S (Fig. 2C). Moreover, CF03-L produced significantly more biofilm than ATCC 29213 and Newman in presence of HQNO, revealing that the amplitude of the response of normal strains to HQNO may individually differs (Fig. 2C). Interestingly, an overnight exposure to 10 μg/ml of HQNO resulted in a significant increase in biofilm production (*P *< 0.05) for strain Newman, CF03-L and CF1A-L even after sub-culturing strains in HQNO-free medium (data not shown). This indicates that an exposure of *S. aureus *to HQNO may result in a sustained increase in biofilm production. Overall, these results suggest that HQNO increases biofilm production in normal *S. aureus *strains and that the sustained effect of HQNO on biofilm production in subsequent HQNO-free medium may result from the relative increase in the sub-population of SCVs which are good biofilm producers.

### SigB is involved in HQNO-mediated emergence of SCVs and biofilm production

**Figure 3:**
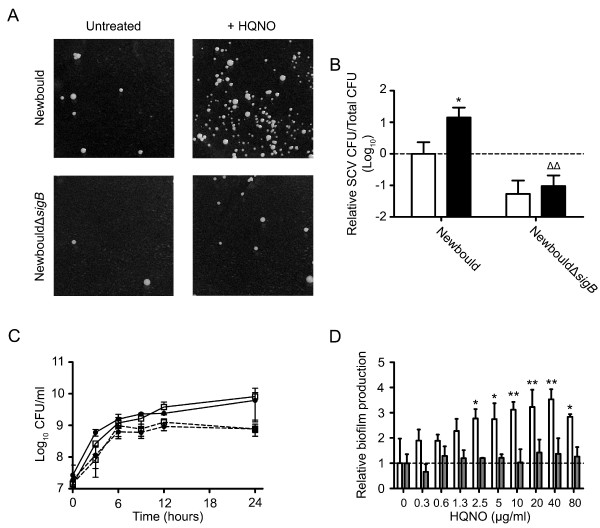
**SigB is involved in HQNO-mediated emergence of SCVs and biofilm production**. (A) Pictures show SCV colonies grown on agar containing a selective concentration of gentamicin following or not an overnight exposure to 10 μg/ml of HQNO for strains Newbould and NewbouldΔ*sigB*. (B) Relative number of SCV CFUs recovered after 18 h of growth for strains Newbould and NewbouldΔ*sigB *in the presence (black bars) or not (open bars) of 10 μg HQNO/ml. Results are normalized to unexposed Newbould (dotted line). Data are presented as means with standard deviations from at least three independent experiments. Significant differences between unexposed and HQNO-exposed conditions (*, *P *< 0.05), and between strains in the same experimental condition (Δ, *P *< 0.05) were revealed by a one-way ANOVA with tuckey's post test. (C) Growth curves of Newbould (□) and NewbouldΔsigB (●) exposed (dotted lines) or not (solid lines) to 10 μg/ml of HQNO. (D) Relative biofilm formation as a function of the concentration of HQNO for strains Newbould (open bars) and NewbouldΔ*sigB *(grey bars). Results are normalized to the unexposed condition for each strain (dotted line). Data are presented as means with standard deviations from two independent experiments. Significant differences between Newbould and NewbouldΔ*sigB *for each concentration of HQNO are shown (*, *P *< 0.05; **, *P *< 0.01; two-way ANOVA with bonferroni's post test).

Strains Newbould and NewbouldΔ*sigB *were used to determine whether SigB is involved in the emergence of SCVs and biofilm production under an exposure to HQNO. Fig. 3A illustrates the ability of HQNO (10 μg/ml, overnight) to favor the emergence of the SCV phenotype only in a *sigB*^+ ^background. HQNO significantly increased the presence of SCVs in strain Newbould, but not in NewbouldΔ*sigB *(Fig. 3B). This result was confirmed with strains SH1000 and 8325-4 (data not shown), which are isogenic strains with a functional and dysfunctional SigB system, respectively [36]. Fig. 3C demonstrates that the presence of HQNO significantly inhibits the growth of both Newbould and NewbouldΔ*sigB *(*P *< 0.05 at 24 h of growth for both; two-way ANOVA followed by a Bonferroni's post test). However, the ability of HQNO to increase biofilm formation was observed with strain Newbould, but not with NewbouldΔ*sigB *(Fig.3D). These results suggest that, even if the inhibition of growth caused by HQNO is not influenced by SigB (Fig. 3C), HQNO-mediated emergence of SCVs and biofilm production is triggered by a SigB-dependent mechanism (Fig. 3D).

### SigB and *agr *activities are modulated by an exposure to HQNO

**Figure 4:**
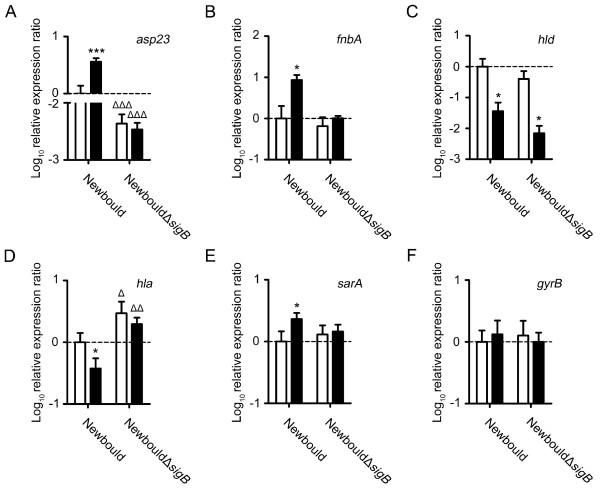
**SigB and *agr *activities are modulated by an exposure to HQNO**. Relative expression ratios for the genes *asp23 *(A), *fnbA *(B), *hld *(C), *hla *(D), *sarA *(E) and *gyrB *(F) were evaluated by qPCR for strains Newbould and NewbouldΔ*sigB *grown to the exponential phase in the presence (black bars) or in the absence (open bars) of 10 μg/ml of HQNO. Results are normalized to unexposed Newbould (dotted line). Data are presented as means with standard deviations from at least three independent experiments. Significant differences between the unexposed and HQNO-exposed conditions (*, *P *< 0.05; ***, *P *< 0.001) and between Newbould and NewbouldΔ*sigB *for the same experimental condition (Δ, *P *< 0.05; ΔΔ, *P *< 0.01; ΔΔΔ, *P *< 0.001) were revealed by one-way ANOVA followed by the tuckey's post test.

Fig. 4 shows qPCR measurements of the expression of the genes *asp23*, *fnbA*, *hld *(RNAIII), *hla*, *sarA *and *gyrB *at the exponential growth phase for strains Newbould and NewbouldΔ*sigB *exposed or not to HQNO. The expression of *asp23 *and *fnbA *was evaluated in order to verify the hypothesis that SigB is activated during HQNO exposure. The gene *asp23 *is a well-known marker for SigB activity as for the gene *fnbA*, although the transcription of the latter is not exclusively influenced by SigB [15,19,22,37]. Fig. 4A and 4B show that HQNO at 10 μg/ml induced SigB activity in strain Newbould, as revealed by significant increases of *asp23 *and *fnbA *expression. The effect of HQNO on the expression of *asp23 *and *fnbA *was further confirmed with the sequenced strain Newman (data not shown). These results suggest that SigB activity is increased by HQNO.

The activity of the *agr *system is known to be reduced in SCVs [15,38-41]. We have thus hypothesized that HQNO exposure would repress the *agr *quorum-sensing system due to the general suppression of growth toward normal strains (likely mediated through the inhibition of the electron transport chain by HQNO [42]) but also due to the overall emergence of the SCV sub-population as seen in Fig. 1. Indeed as expected, Fig. 4C shows that exposure of Newbould and NewbouldΔ*sigB *to HQNO significantly repressed the expression of *hld *(the effector of the *agr *system).

With the increased in SigB activity and the reduced expression of *agr *observed under exposure to HQNO, it was also justified to measure the expression of the α-hemolysin gene *hla *which can be influenced by both *agr *and SigB [36,43]. *hla *was only significantly repressed in Newbould and not in NewbouldΔ*sigB *by the presence of HQNO (Fig. 4D). Furthermore, the expression of *hla *was, in both exposed and unexposed conditions, significantly increased in NewbouldΔ*sigB *in comparison to Newbould, which confirms the negative influence of SigB on *hla *expression [36]. These results show that the expression of *hla *is reduced by HQNO and that the influence of SigB on *hla *expression under HQNO exposure seems to be predominant over the *agr *system.

The expression level of *sarA *was also measured because of its partial dependency on SigB for expression [22,23], and its roles in the regulation of virulence factors expression [24] and in biofilm formation [29]. Fig. 4E shows that *sarA *expression is significantly induced by HQNO in strain Newbould but not in NewbouldΔ*sigB*. The specificity of the observed modulations in gene expression was validated by monitoring the impact of HQNO on the expression of the housekeeping gene *gyrB*. The expression of *gyrB *was not modulated in the different conditions tested (Fig. 4F). These results suggest that HQNO induces the expression of *sarA *by a SigB-dependent mechanism.

Overall, these results suggest that exposure of *S. aureus *to HQNO reproduces the transcriptional signature found in SCVs [12,15,19,20,41] and stimulates biofilm production by having opposite effects on the activity of SigB (up) and *agr *(down) as well as on the expression of *sarA *(up by a SigB-dependent mechanism).

### *P. aeruginosa *stimulates biofilm formation and increases the activity of SigB of a *S. aureus *CF isolate

**Figure 5:**
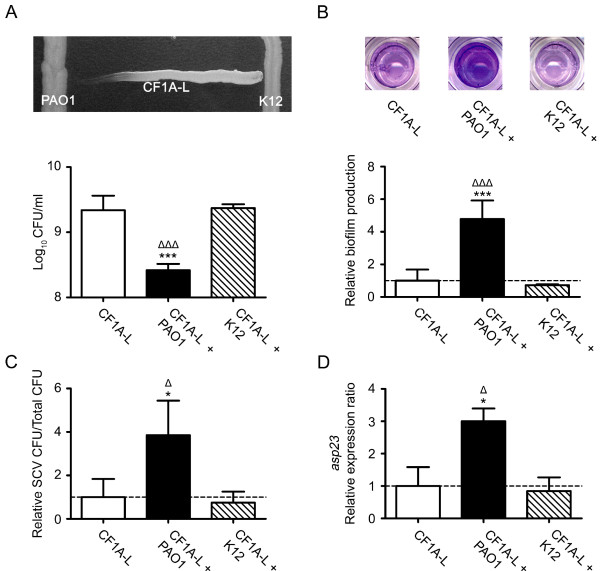
***P. aeruginosa *stimulates biofilm formation and increases the activity of SigB of a *S. aureus *CF isolate**. (A) CFU/ml recovered after 48 h of growth of CF1A-L (open bar) and CF1A-L in the presence of supernatants from overnight cultures of *P. aeruginosa *PAO1 (black bar) or of *E. coli *K12 (hatched bar). The picture shows the specific inhibitory effect of *P. aeruginosa *on the growth of *S. aureus*. (B) Relative biofilm production by CF1A-L grown in the presence of supernatants from overnight cultures of *P. aeruginosa *or *E. coli*. Pictures show the biofilm formation of CF1A-L in the absence or in the presence of culture supernatants of *P. aeruginosa *or *E. coli *as detected by crystal violet staining. (C) Relative number of SCV CFUs recovered after 6 h of growth for *S. aureus *CF1A-L in co-culture with PAO1 or K12 as determined using the double chamber co-culture model. (D) Relative expression ratios for the gene *asp23 *were evaluated by qPCR for CF1A-L in co-culture with PAO1 or K12. For B, C and D, results are normalized to unexposed CF1A-L (dotted line). Data are presented as means with standard deviations from three independent experiments. Significant differences between unexposed CF1A-L and the exposed conditions (*, *P *< 0.05; ***, *P *< 0.001) and between CF1A-L exposed to PAO1 or K12 (Δ, *P *< 0.05; ΔΔΔ, *P *< 0.001) were revealed by one-way ANOVA followed by the tuckey's post test.

In order to ascertain that the effect of HQNO on *S. aureus *is representative of what may happen when *P. aeruginosa *and *S. aureus *are in close proximity during a co-infection, we conducted experiments in which *S. aureus *was exposed to supernatants from overnight cultures of *P. aeruginosa *as well as experiments using a double chamber co-culture model. We used the *E. coli *strain K12 in control experiments to ensure that the observed effect was specific to *P. aeruginosa *and was not only caused by the close proximity of a Gram-negative bacterium or non specific alterations of the growth medium. We used *E. coli *because it is known that this bacterium does not produce HQNO (E. Déziel, unpublished data). Fig.5A shows that *P. aeruginosa *PAO1 inhibits the growth of the *S. aureus *strain CF1A-L whereas this phenomenon was not observed with *E. coli *K12. The supernatant collected from an overnight culture of PAO1 significantly inhibited the growth of *S. aureus*. This growth inhibition was accompanied by a significant increase in biofilm production (Fig. 5B). Fig. 5C shows that when *S. aureus *CF1A-L was co-cultured with PAO1 for 6 h, significantly more SCVs were recovered than that seen when the co-culture was done with *E. coli *K12. Of striking interest, the co-cultivation of *S. aureus *CF1A-L with *P. aeruginosa *PAO1 specifically and significantly increased the expression of *asp23*. These results confirm that *P. aeruginosa *has the potential to specifically inhibit the growth, stimulate biofilm production, favor the emergence of the SCV phenotype and increase the activity of SigB in non-SCV *S. aureus *strains.

### HQNO from *P. aeruginosa *stimulates *S. aureus *biofilm production by a SigB-dependent mechanism

**Figure 6:**
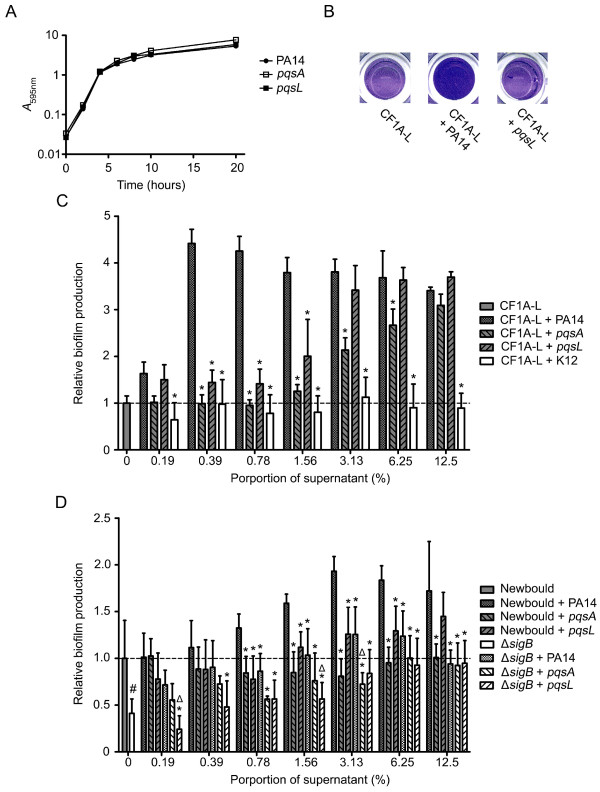
**HQNO from *P. aeruginosa *stimulates biofilm production of *S. aureus *strains by a SigB-dependent mechanism**. (A) Growth curves of *P. aeruginosa *strain PA14 and the *pqsA *and *pqsL *mutants. (B) Pictures show relative biofilm production of CF1A-L in the absence or in the presence of supernatants from overnight cultures of *P. aeruginosa *PA14 or the *pqsL *mutant as determined by crystal violet staining. (C) Relative biofilm production by *S. aureus *CF1A-L as a function of the proportion of supernatant from overnight cultures of *P. aeruginosa *PA14, the *pqsA *mutant, the *pqsL *mutant or *E. coli *K12. Results are normalized to unexposed CF1A-L (dotted line). Significant differences between CF1A-L+PA14 and the other conditions for each proportion of supernatant are shown (*, *P *< 0.05; two-way ANOVA with Bonferroni's post test). (D) Relative biofilm production by *S. aureus *strains Newbould and NewbouldΔ*sigB *as a function of the proportion of supernatant from overnight cultures of *P. aeruginosa *PA14, the *pqsA *or the *pqsL *mutant. Significant differences between Newbould + PA14 and the other conditions for each proportion of supernatant (*, *P *< 0.05; two-way ANOVA with Bonferroni's post test), and between NewbouldΔ*sigB *+ PA14 and Newbould Δ*sigB *+ the *pqsA *or the *pqsL *mutant (Δ, *P *< 0.05; two-way ANOVA with Bonferroni's post test) are shown. The significant difference between untreated Newbould and NewbouldΔ*sigB *is also shown (#, *P *< 0.05; unpaired *t*-test). Data are presented as means with standard deviations from at least three independent experiments.

We used the *pqsA *and *pqsL *mutants derived from *P. aeruginosa *PA14 to further confirm the specific effect of HQNO on biofilm production by *S. aureus*. The *pqsA *mutant does not produce any 4-hydroxy-2-alkylquinolines (HAQs) at all [44,45], whereas the *pqsL *mutant is specifically altered in HQNO biosynthesis [46]. Thus, we have used both *pqsA *and *pqsL *mutants in order to distinguish the global impact of all *P. aeruginosa *HAQs from the specific impact of HQNO on biofilm production by *S. aureus*. Fig. 6A shows that the growth of the *pqsA *and *pqsL *mutants is not impaired compared to that of the parental strain PA14, thus excluding variations in supernatant composition caused by differences in growth rates among strains. Fig. 6B shows that the supernatant from an overnight culture of *P. aeruginosa *PA14 stimulates biofilm production by *S. aureus *CF1A-L in comparison to the supernatant from the *pqsL *mutant (specific HQNO-minus strain). The effect of different doses of supernatants from overnight cultures of *P. aeruginosa *PA14, the *pqsA *mutant, the *pqsL *mutant or *E. coli *K12 on biofilm production by *S. aureus *CF1A-L is shown in Fig. 6C. While supernatants from both mutants significantly induced less biofilm production in comparison to PA14, this attenuated effect was more pronounced for the *pqsA *mutant (negative for the production of all HAQs) than the *pqsL *mutant. This result can be explained by the fact that other HAQs secreted by *P. aeruginosa*, although less potent than HQNO, can also have a growth-inhibitory activity against *S. aureus *[47]. Noteworthy, all three strains of *P. aeruginosa *stimulated biofilm production in comparison to *E. coli*, suggesting that other *P. aeruginosa *exoproducts can indeed stimulate biofilm production by *S. aureus*.

Fig. 6D confirms that HQNO from the supernatant of strain PA14 stimulates biofilm production by a SigB-dependent mechanism. The increase in biofilm production observed when *S. aureus *Newbould is in contact with the supernatant from PA14 is significantly higher than that seen with supernatants from the *pqsA *and *pqsL *mutants. Surprisingly, both mutants did not significantly stimulate biofilm production by Newbould as that observed for CF1A-L, suggesting that differences between *S. aureus *strains may exist in respect to their response to the presence of non-HQNO exoproducts. As expected, biofilm production by NewbouldΔ*sigB *in contact with supernatants from the three *P. aeruginosa *strains was significantly inferior to that observed using the PA14 supernatants with strain Newbould. Moreover, supernatants from PA14 generally did not significantly stimulate biofilm production by NewbouldΔ*sigB *in comparison to supernatants from *pqsA *and *pqsL *mutants, which confirms that SigB is involved in HQNO-mediated *S. aureus *biofilm production. Overall, the results of this section support the hypothesis that HQNO from *P. aeruginosa *stimulates biofilm production by *S. aureus *through a SigB-dependent mechanism.

## Discussion

We found that the *P. aeruginosa *exoproduct HQNO increases the production of biofilm by *S. aureus*. The effects on biofilm production, as well as on growth, were only seen on normal strains whereas the already high biofilm formation and slow growth rate of SCVs were not altered by the presence of HQNO. It is known that HQNO specifically inhibits electron transport in Gram-positive bacteria [42] and its immediate action toward normal strains is likely to reduce ATP production and growth to a similar level to that observed in SCVs. Interestingly, our results also indicate that HQNO provokes a sustained stimulatory effect on the production of biofilms by *S. aureus*. We indeed found that a pre-treatement of *S. aureus *with HQNO still led to a subsequent increase in biofilm formation even after HQNO removal. This sustained effect is probably associated with the increased proportion of the sub-population of SCVs resulting from HQNO exposure. An exposure of *S. aureus *to HQNO may thus, in addition to its immediate effect, favor the emergence of SCVs having a long-term impact on biofilm formation.

Aminoglycosides are also known to favor the emergence of SCVs [12] and are often used in CF patient care [1]. Interestingly, a synergistic effect between HQNO and tobramycin for the formation of *S. aureus *SCVs was previously observed by Hoffmann *et al. *[2]. It is thus possible that the administration of aminoglycosides to CF patients co-infected with both *S. aureus *and *P. aeruginosa *further increases the formation of biofilm by *S. aureus*. Besides, it is well known that the abnormal function of the CF transmembrane conductance regulator (CFTR) protein in CF patients has profound consequences on the airway physiology and it will be of great interest to determine whether other parameters related to the CF airways influence the emergence of SCVs and the production of biofilms by *S. aureus*. The expression of virulence factors in *S. aureus *is indeed controlled by diverse and complex regulatory networks in a time- and environment-dependent manner, being influenced for example by ionic forces, pH and O_2 _[48]. Consequently, it is likely that *S. aureus *specifically responds to the particular environment of CF airways. Whether this response is SigB-dependent and will lead to the emergence of SCVs and biofilm production remains to be determined.

Naturally-occurring mutations altering the activity of virulence regulators in *S. aureus *have been previously reported [36,49-52]. Our results suggest that the inactivation of *sigB *will importantly influence the outcome of the HQNO-mediated interaction between *P. aeruginosa *and *S. aureus*. We are currently studying *S. aureus *isolates from CF patients co-infected with *P. aeruginosa *which are not influenced by the presence of *P. aeruginosa*. This, in addition to the observation that differences between *S. aureus *strains exist relative to their response to HAQs (Fig. 6C and 6D), suggest that *S. aureus *strains isolated from CF patients may adapt or evolve toward a long-term coexistence with *P. aeruginosa*. Whether this involves mutations in *sigB *or any other genes encoding regulators is now under investigation and will greatly help to understand the dynamic behavior and the adaptation of *S. aureus *in response to the CF airway environment as well as to the presence of *P. aeruginosa*.

The effect of HQNO on the regulators SarA, *agr *and SigB suggests that several virulence factors should be influenced by the presence of HQNO. A concomitant activation of SigB and repression of *agr *should result in the up-regulation of a variety of cell-surface proteins (such as FnBA) involved in adhesion to host tissues, and in the repression of several exotoxins (such as α-hemolysin, Hla) and proteolytic enzymes [22,25]. It is then tempting to speculate that the presence of HQNO will prevent *S. aureus *from disseminating and will rather favor tissue colonization, biofilm production and invasion of host cells. It has indeed been suggested that *S. aureus *FnBPs mediates cellular invasion [53,54] whereas the capacity of the bacterium to remain intracellular is helped by the repression of *hla *[55]. Accordingly, we showed that an exposure of *S. aureus *to HQNO up-regulates the expression of *fnbA *and represses the expression of *hla*. However, whether or not HQNO and *P. aeruginosa *increase the invasion of host cells by *S. aureus *remains to be confirmed. Interestingly, O'Neil *et al. *[32] have recently demonstrated that the FnBPs are also involved in the *ica-*independent mechanism of biofilm formation. It is thus possible that FnBPs are directly responsible for the observed HQNO-mediated SigB-dependent increase in biofilm production and, more specifically, FnBPA which is under the control of SigB for expression [15,19,22,37]. As such, the FnBPs would represent the main effectors for both biofilm formation and cellular invasion in *S. aureus *SCVs.

HQNO may be one of several bacterial exoproducts influencing *S. aureus *during polymicrobial infections. Our results and those of Machan *et al. *[47] suggest that other HAQs may also affect *S. aureus*, although not as efficiently as HQNO. Moreover, it is known that other *P. aeruginosa *exoproducts such as pyocyanin have an inhibitory activity against the electron transport chain of *S. aureus *[13]. Loss of pyocyanin production has been associated with mutations in the *pqsA-E *genes [45,56], which may provide an additional explanation for the different effects of the *pqsA *and *pqsL *mutants we have observed on the growth (data not shown) and biofilm formation of *S. aureus *(Fig. 6C). Furthermore, Qazi *et al. *[7] found that an *N-*acyl-homoserine-lactone from *P. aeruginosa *antagonizes quorum sensing and virulence gene expression in *S. aureus*. More precisely, it was shown that the 3-oxo-C_12_-HSL interacts with the cytoplasmic membrane of *S. aureus *and down-regulates both *sarA *and *agr *expression. Although we also observed here a down-regulation of *agr*, the HQNO-mediated up-regulation of *sarA *suggests further complexity in the response of *S. aureus *to *P. aeruginosa *exoproducts. It is possible that the outcome of the *S. aureus-P. aeruginosa *interaction is dependent on the amount and the types of exoproducts secreted by the specific strain of *P. aeruginosa *interacting with *S. aureus*.

It should be kept in mind that the diversity of diffusible bacterial signals and their role in interspecies communication are just beginning to be appreciated [9] and that potentially numerous other bacterial species may be interacting with *S. aureus*, especially during infectious diseases. It is then likely that *S. aureus *interacts with other bacterial genus than *Pseudomonas *during infection of the airways of CF patients. As an example, the CF pathogen *Burkholderia cepacia *also produces *N*-acylhomoserine lactones [57] and some *Burkholderia *species are able to synthesize HAQ analogues [58]. Nevertheless, the observation that *P. aeruginosa *favors the emergence of SCVs and biofilm production by *S. aureus *is likely to have a significant clinical impact. The clinical consequences may actually surpass the previously anticipated formation of aminoglycoside-resistant SCVs by Hoffman *et al*. [2]. Persistence of bacteria in chronic infections has been associated with biofilm production [1,59] and biofilms are known to confer protection from host defenses and antibiotic treatments at large [34,60]. In the cystic fibrosis context, where obstructive infections worsen the health prognosis of patients, the clinical significance of biofilm production by normal *S. aureus *and SCV strains will need to be further investigated.

## Conclusions

This study strongly supports the hypothesis that *P. aeruginosa *influences the pathogenicity of *S. aureus *by producing HQNO, which favors the acquisition of the SCV phenotype through the activation of the stress- and colonization-related *S. aureus *alternative sigma factor B. Although several *P. aeruginosa *exoproducts may potentially influence *S. aureus*, our observations with pure HQNO were confirmed and supported by experiments using whole supernatants from two *P. aeruginosa *strains as well as mutants unable to produce HQNO. Considering that biofilms and SCVs are both suspected to play a role in chronic infections of CF airways, the observation that *P. aeruginosa *increases the emergence of SCVs and biofilm formation by *S. aureus *may influence the patient health prognosis. New therapeutic strategies should aim at preventing interspecies interactions and the development of specific phenotypes such as biofilm-producing SCVs in order to reduce the likelihood of chronic infections.

## Methods

### Bacterial strains and growth conditions

The relevant characteristics of the strains used in this study are shown in Table 1. *Staphylococcus aureus *ATCC 29213, Newman and Newbould were used as representatives of prototypical control strains. NewbouldΔ*sigB *and Newbould*hemB*, in which the genes *sigB *or *hemB *had been disrupted by the *ermA *cassette [15,17], were used to evaluate the importance of SigB in a prototypical background and to generate a stable SCV, respectively. CF03-L/CF03-S, CF07-L/CF07-S and CF1A-L/CF1D-S are related pairs of strains co-isolated from CF patients, which respectively have a normal and a SCV phenotype. The genetic relatedness of each strain among the pairs was confirmed by the analysis of multiple loci with a variable number of tandem repeats (see below). Except where otherwise stated, *S. aureus *strains were grown in brain heart infusion (BHI) broth (BD, ON, Canada). The use of BHI to study our SCV strains as well as in the experiments involving quantification of SCVs is validated in the Additional file 1. *Pseudomonas aeruginosa *PAO1 [61], PA14 [62], the PA14-derived *pqsA *and *pqsL *mutants [44,46] and *Escherichia coli *K12 were grown in trypticase soy broth (TSB) (BD, ON, Canada).

**Table 1:** Bacterial strains used in this study

Strains	Relevant characteristics	Auxotrophism	References
*S. aureus *strains			
ATCC 29213	Laboratory strain, normal	-	-
Newman ATCC 25904	Laboratory strain, normal	-	-
Newbould ATCC 29740	Laboratory strain, normal	-	-
NewbouldΔ*sigB*	Newbould Δ*sigB*::*emrA*; Erm^R^	-	[15]
Newbould*hemB*	Newbould *hemB*::*ermA*; Erm^R^	Hemin	[17]
CF03-S	SCV strain isolated from a CF patient	Menadione	[15]
CF03-L	Normal strain co-isolated with CF03-S	-	This study
CF07-S	SCV strain isolated from a CF patient	Menadione	[15]
CF07-L	Normal strain co-isolated with CF07-S	-	This study
CF1D-S	SCV strain isolated from a CF patient	Unknown	This study
CF1A-L	Normal strain co-isolated with CF1D-S	-	This study
			
*P. aeruginosa *strains			
PAO1	Laboratory strain	-	[61]
PA14	Clinical strain, Rif^R^	-	[62]
*pqsA*	PA14 *pqsA*::Tn*phoA*; Rif^R^, Km^R^	-	[44]
*pqsL*	PA14 Δ*pqsL*; Rif^R^	-	[46]
			
*E. coli *strains			
K12	Laboratory strain	-	-

### Multiple-locus variable-number of tandem repeat analysis (MVLA) of strains co-isolated from CF patients

The relatedness of each of the co-isolated strains within the pairs CF03-L/CF03-S, CF07-L/CF07-S and CF1A-L/CF1D-S was confirmed by MVLA as described by Sabat *et al. *[63]. The strains of each pair had identical MVLA patterns.

### Growth curves

*S. aureus *overnight cultures were used at an *A*_595 nm _of 0.1 to inoculate BHI broths supplemented or not with 10 μg/ml of HQNO (Axxora, CA, USA). Cultures were then incubated at 35°C/225 RPM and samples were taken at different time points for determination of CFU by spreading 10-fold dilutions on trypticase soy agar (TSA) plates (BD, ON, Canada). Plates were incubated at 35°C for 24 and 48 h for normal and SCV strains, respectively. For the growth curves of *P. aeruginosa *PA14 and the *pqsA *and *pqsL *mutants, overnight cultures were used to inoculate TSB. Cultures were then incubated at 35°C/225 RPM and samples were taken at specified time points in order to evaluate their turbidity at *A*_595 nm_.

### Quantification of SCVs

We have quantified SCVs by taking advantage of their reduced susceptibility to aminoglycosides as described elsewhere with few modifications [20,64,65]. A 1:100 dilution of overnight broth cultures was used to inoculate BHI broths supplemented or not with 10 μg/ml of HQNO. Cultures were incubated 18 h and then adjusted to an *A*_595 nm _of 2.0 in PBS at 4°C. Determination of SCV CFUs was done by serial dilution plating. SCV counts were obtained by plating on TSA containing gentamicin (Sigma-Aldrich, ON, Canada) at 4 μg/ml followed by an incubation of 48 h at 35°C. As shown in Additional file 2, this concentration of gentamicin is selective for SCVs as it allows the growth of SCVs, but not that of normal strains. The frequency of SCVs is defined as the number of SCVs per total CFU counts on antibiotic-free TSA. The pinpoint colonies detected by this gentamicin-plate method were confirmed to be SCVs by streaking several of them on TSA plates (See Additional file 3). We have also evaluated the auxotrophism (as described below) of several HQNO-induced SCVs generated from strains CF1A-L and CF07-L in order to further validate the ability of this technique to detect typical SCVs (see Additional file 4).

### Antibiotic susceptibility

The minimal inhibitory concentrations (MICs) of gentamicin for all strains were determined by a broth microdilution technique, following the recommendations of the Clinical and Laboratory Standards Institute (CLSI) guidelines [66], except that the incubation period was extended to 48 h and that the medium used was BHI in order to allow SCVs to reach maximal growth.

### Auxotrophism of SCVs

In the context of SCVs, auxotrophism is defined as the requirement of specific compounds in order to regain a normal growth phenotype [41]. An agar diffusion method was used to characterize the auxotrophism of SCVs using hemin or menadione (10 μg each/well) on an inoculated Mueller-Hinton agar (MHA) plate. Thymidine at 1.5 μg/well was also tested as previously described [67]. Auxotrophy for specific supplements was detected by a zone of normal growth surrounding the well after 18 h of incubation at 35°C. The photography of the Additional file 5 shows the normal growth of Newbould*hemB *in proximity of a well loaded with hemin as an example of a positive auxotrophism result.

### Preparation of supernatants from *P. aeruginosa *and *E. coli *strains

Overnight cultures were used to inoculate TSB at a dilution of 1:100. Cultures were then incubated 20 h at 35°C/225 RPM before collecting the culture supernatants by centrifugation. Similar culture conditions were previously shown to allow maximal production of HQNO by *P. aeruginosa *PAO1 [68]. The supernatants were then filter-sterilized using 0.22 μ pore size (Millipore, MA, USA) and used immediately. The sterility of the supernatants was confirmed by plating samples on TSA plate.

### Biofilm formation

For studying the effect of HQNO on biofilm production by *S. aureus*, three colonies grown on blood agar plates were used to inoculate BHI broths containing 0.25% glucose with or without 10 μg/ml of HQNO and cultures were incubated for 18 h. These cultures were used to adjust an appropriate volume of BHI-0.25% glucose to 0.5 Mcfarland for transfer into wells of a flat-bottom polystyrene microtiter plate containing half volume of the same medium with or without HQNO (final concentration 10 μg/ml). For experiments evaluating the effect of culture supernatants from *P. aeruginosa *and *E. coli *on *S. aureus *biofilm production, a *S. aureus *0.5 Mcfarland suspension was prepared in BHI-0.5% glucose and transferred into wells of a microtiter plate containing half the volume of the supernatant to be tested. The plates were incubated at 35°C for 48 h. The supernatant was then discarded and the wells were delicately washed three times with 200 μl of PBS. The plates were dried, stained for 30 min with crystal violet, washed twice with 200 μl of water and allowed to dry again. A volume of 200 μl of 95% ethanol was added to each well and plates were incubated at room temperature for 1 h with frequent agitation. The absorbance of each well was then measured at 560 nm using a plate reader (Bio-Tek Instruments). The biofilm formation of each culture tested was evaluated in four replicates. The *A*_560 nm _values (non-normalized data) representing the biofilm production for each of the strains used in Fig. 2 can be seen in the Additional file 6.

### Quantitative PCR (qPCR)

In order to evaluate the effect of HQNO (10 μg/ml) on *S. aureus *gene expression, overnight cultures were used to inoculate broth at an *A*_595 nm _of 0.1. Bacteria were then grown until the unexposed control culture reached an *A*_595 nm_between 0.9 and 1.0. Bacteria were collected and treated with RNAprotect (QIAGEN, ON, Canada). RNA was extracted from the cell pellets after treatment with lysostaphin (Sigma-Aldrich) (200 μg/ml, 1 h) using the RNeasy Mini kit and the RNase-free DNase set (QIAGEN). A second DNase treatment was also done with the DNA-free kit (Applied Biosystems/Ambion, CA, USA). One μg of total RNA was reverse transcribed with 0.5 mM deoxynucleotide phosphate, 50 ng of random hexamers and 200 U of Invitrogen Superscript II reverse transcriptase, according to the manufacturer's recommendations (Invitrogen, ON, Canada). RNA was hydrolyzed and the cDNAs were purified with the QIAquick PCR purification kit (QIAGEN). One microliter of the cDNA preparation was amplified on the Stratagene MX3000P Real-Time PCR instrument with the Jump Start Taq DNA polymerase (Sigma-Aldrich), SYBR Green and 100 nM of the following primers:

*asp23*-RT-FWD 5'-TCGCTGCACGTGAAGTTAAA-3',

*asp23*-RT-REV 5'-CAGCAGCTTGTTTTTCACCA-3',

*fnbA268*-RT-FWD 5'-ACAAGTTGAAGTGGCACAGCC-3',

*fnbA341*-RT-REV 5'-CCGCTACATCTGCTGATCTTGTC-3',

*hld*-RT-FWD 5'-TAATTAAGGAAGGAGTGATTTCAATG-3'

*hld*-RT-REV 5'-TTTTTAGTGAATTTGTTCACTGTGTC-3'

*hla*-RT-FWD 5'-AATGAATCCTGTCGCTAATGCCGC-3'

*hla*-RT-REV 5'-CTGAAGGCCAGGCTAAACCACTTT-3'

*sarA*-RT-FWD 5'-CAAACAACCACAAGTTGTTAAAGC-3'

*sarA*-RT-REV 5'-TGTTTGCTTCAGTGATTCGTTT-3'

16SrRNA-RT-FWD 5'- TCGTTTAACACGTTTAGGTTCA-3',

16SrRNA-RT-REV 5'- GAACTGTATCAGTTGGTTTCGCAC-3',

*gyrB*-RT-FWD 5'-GGTGCTGGGCAAATACAAGT-3',

*gyrB*-RT-REV 5'-TCCCACACTAAATGGTGCAA-3'.

Reaction mixtures were denatured for 10 min at 95°C, followed by 35 cycles of 30 s at 95°C, 1 min at 60°C and 1 min 30 s at 72°C. Dissociation and standard curves were obtained to insure the specificity and the efficiency of reactions. cDNA synthesis reactions without reverse transcriptase were also routinely carried out. The relative expression ratios were calculated by using the cycle threshold (C_t_) of the 16S RNA or *gyrB *of each condition as the calibrator (*n*-fold expression = 2^-ΔCt^, where ΔC_t _represents the difference between the C_t _of the gene studied and the C_t _of the 16S RNA or *gyrB *for each condition).

### Double chamber co-culture model

Overnight cultures of *S. aureus, E. coli *and *P. aeruginosa *in TSB were used to inoculate bottom (*S. aureus, E. coli *or *P. aeruginosa*) or top (*S. aureus*) chambers of 0.4-μm pore polycarbonate membrane inserts (Transwell [Corning, MA, USA]). *S. aureus *was inoculated at an *A_595 nm _*of 0.01, whereas *P. aeruginosa *or *E. coli *were inoculated at an *A_595 nm _*of 0.1. The cultures were incubated at 35°C/80 RPM for 6 h and samples were taken for SCV enumeration and total CFU counts as well as for RNA extraction. No bacterial cross-contamination was detected by culture plating up to at least 9 h of incubation.

### Statistical analysis

One-way analysis of variance followed by Dunnett's multiple comparisons test or Tukey's multiple comparisons test were used when several conditions or strains were compared at the same time whereas unpaired *t*-tests were used when only two conditions were compared. Two-way ANOVA with Bonferroni's post tests were used to compare the response of different strains and/or different conditions as a function of the concentration of HQNO or bacterial culture supernatants. Statistical analyses of qPCR data were done on mean ΔC_*t*_. CFU counts or SCV frequencies were transformed in based-10 logarithm values before being used for statistical analyses that were carried out with the GraphPad Prism Software (v.5.00). Statistical tests used for the analysis of each experiment are specified in figure legends.

## Authors' contributions

GM, DLS and AEA carried out the experiments. GM, DLS, ED, AMC, EHF, SM and FM designed and conceived the study. GM and FM wrote the paper. All authors read and approved the final manuscript.

## Supplementary Material

Additional file 1 **Validation of the use of BHI as the growth medium to induce and study SCVs**. (A) Growth curves expressed in absorbance at 595 nm for the strains Newbould, Newbould*hemB*, CF07-L and CF07-S. The growth of Newbould*hemB *and CF07-S was supplemented or not with 5 μg/ml of hemin and 1 μg/ml of menadione, respectively. Results show that SCVs present their slow-growth phenotype in BHI unless supplemental hemin or menadione is added to the broth. (B) Pictures of colonies from strains Newbould, Newbould*hemB*, CF07-L and CF07-S grown on BHI agar for 16 hours. Results show that SCVs retain their slow-growth phenotype on BHIA in comparison to normal strains. (C) Appearance of the colonies obtained from the cultures shown in A at the 12-h time point and plated on Mueller-Hinton agar (MHA) for 36 hours. Results show that normal strains grow efficiently on MHA whereas SCVs taken from BHI broth (cultures shown in A and supplemented or not with hemin or menadione) still present their slow-growth phenotype once plated back on MHA. Click here for file

Additional file 2 **Minimal inhibitory concentrations (MICs) of gentamicin for the studied strains**. Results of this file show that MICs of gentamicin for SCVs are of 8 μg/ml whereas those of normal strains are below 2 μg/ml. Click here for file

Additional file 3 **Appearance of HQNO-induced SCVs selected on gentamicin-containing agar and streaked back on TSA plates**. Pictures are showing CF07-L, CF07-S and HQNO-induced SCVs selected on gentamicin-containing agar and streaked back on TSA plates. The bottom pictures show streaks of three isolated SCVs on TSA plates. Many more SCVs were similarly tested and our results showed that at least 85% of the SCVs isolated from gentamicin plates were keeping their slow-growth phenotype when subsequently grown on TSA without gentamicin. Click here for file

Additional file 4 **Auxotrophism found among HQNO-induced SCVs**. Auxotrophism found among HQNO-induced SCVs generated from the normal cystic fibrosis strains CF07-L and CF1A-L. Click here for file

Additional file 5 **Growth of Newbould*hemB *in proximity of a well loaded with hemin**. Growth of Newbould*hemB *in proximity of a well loaded with hemin as an example of a positive auxotrophism result. The auxotrophism of Newbould*hemB *for hemin is seen by observing normal growth only within the diffusion zone of a well loaded with hemin. Click here for file

Additional file 6 **Non-normalized absorbance values at 560 nm representing biofilm production for each of the strains used in Fig**. 2. Non-normalized absorbance values at 560 nm representing biofilm production for each of the strains used in Fig. 2. Results show that strains vary in their relative production of biofilms but that for each related pairs of normal and SCV strains, SCV counterparts always produce more biofilm than their respective normal strains. Click here for file
