# Supplementary figures and images for: Staphylococcus aureus sigma B-dependent emergence of small-colony variants and biofilm production following exposure to Pseudomonas aeruginosa 4-hydroxy-2-heptylquinoline-N-oxide
Source: BMC Microbiol. 2010 Jan 30;10:33. doi: 10.1186/1471-2180-10-33 (PMC2824698; doi:10.1186/1471-2180-10-33)

A

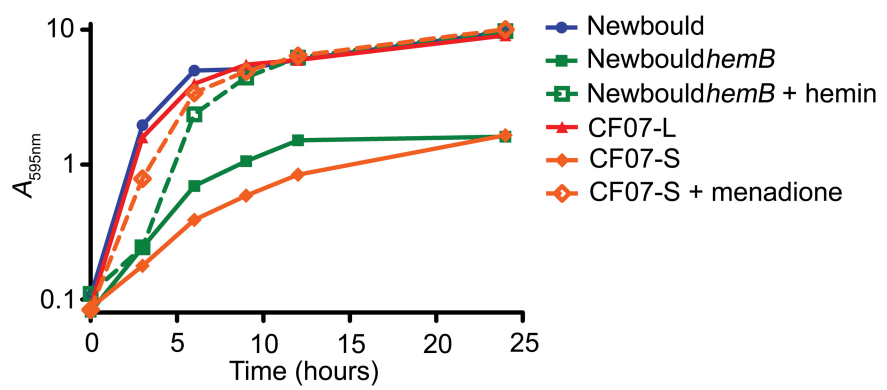

B

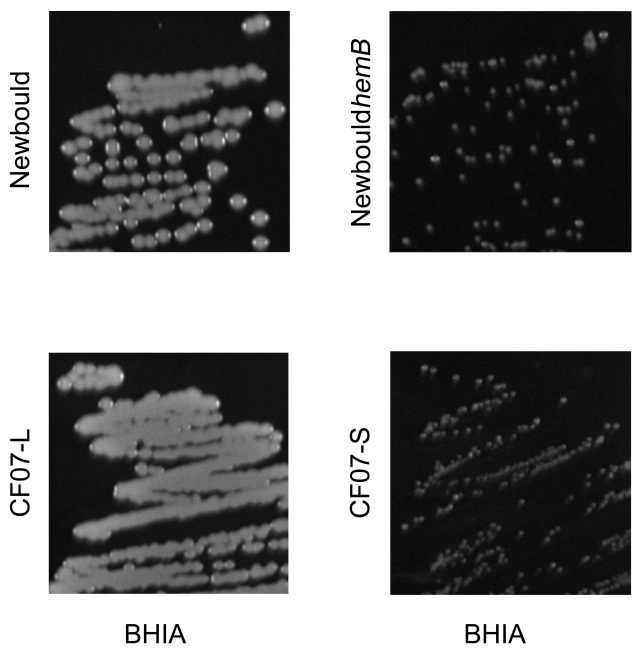

C

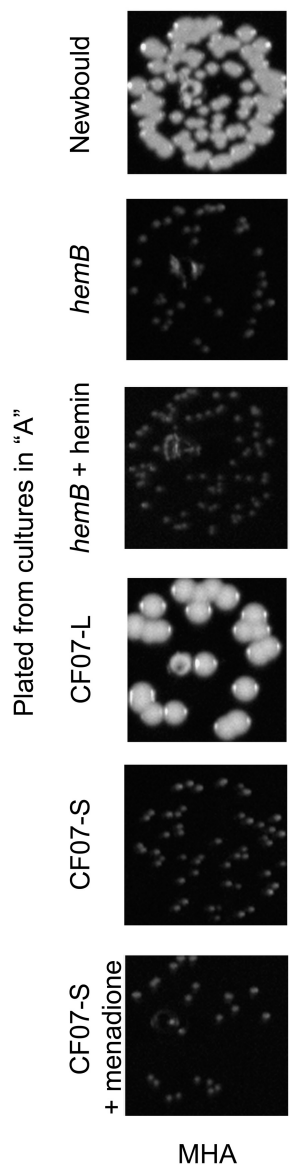

Supplement: Additional file 1 — Validation of the use of BHI as the growth medium to induce and study SCVs. (A) Growth curves expressed in absorbance at 595 nm for the strains Newbould, NewbouldhemB, CF07-L and CF07-S. The growth of NewbouldhemB and CF07-S was supplemented or not with 5 μg/ml of hemin and 1 μg/ml of menadione, respectively. Results show that SCVs present their slow-growth phenotype in BHI unless supplemental hemin or menadione is added to the broth. (B) Pictures of colonies from strains Newbould, NewbouldhemB, CF07-L and CF07-S grown on BHI agar for 16 hours. Results show that SCVs retain their slow-growth phenotype on BHIA in comparison to normal strains. (C) Appearance of the colonies obtained from the cultures shown in A at the 12-h time point and plated on Mueller-Hinton agar (MHA) for 36 hours. Results show that normal strains grow efficiently on MHA whereas SCVs taken from BHI broth (cultures shown in A and supplemented or not with hemin or menadione) still present their slow-growth phenotype once plated back on MHA. [file 1471-2180-10-33-S1.PDF]

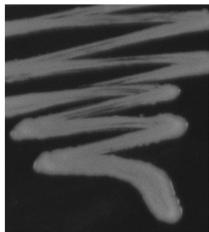

CF07-L

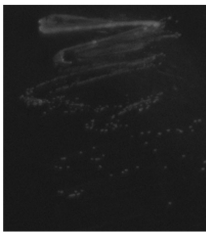

CF07-S

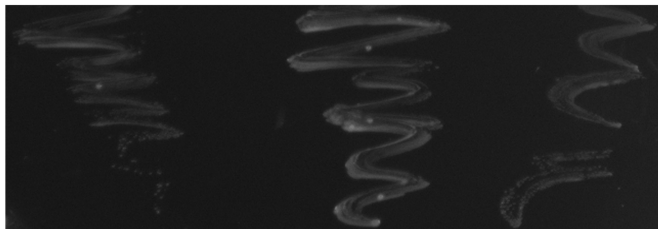

HQNO-induced SCVs  
(from CF07-L)

Additional file 3.

Supplement: Additional file 3 — Appearance of HQNO-induced SCVs selected on gentamicin-containing agar and streaked back on TSA plates. Pictures are showing CF07-L, CF07-S and HQNO-induced SCVs selected on gentamicin-containing agar and streaked back on TSA plates. The bottom pictures show streaks of three isolated SCVs on TSA plates. Many more SCVs were similarly tested and our results showed that at least 85% of the SCVs isolated from gentamicin plates were keeping their slow-growth phenotype when subsequently grown on TSA without gentamicin. [file 1471-2180-10-33-S3.PDF]

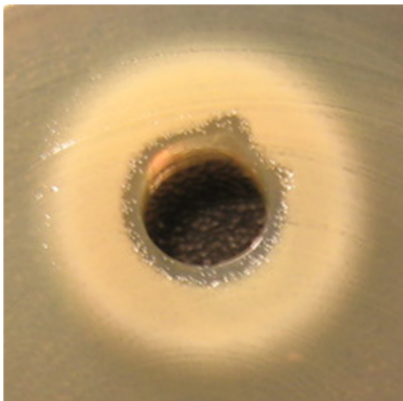

Additional file 5.

Supplement: Additional file 5 — Growth of NewbouldhemB in proximity of a well loaded with hemin. Growth of NewbouldhemB in proximity of a well loaded with hemin as an example of a positive auxotrophism result. The auxotrophism of NewbouldhemB for hemin is seen by observing normal growth only within the diffusion zone of a well loaded with hemin. [file 1471-2180-10-33-S5.PDF]

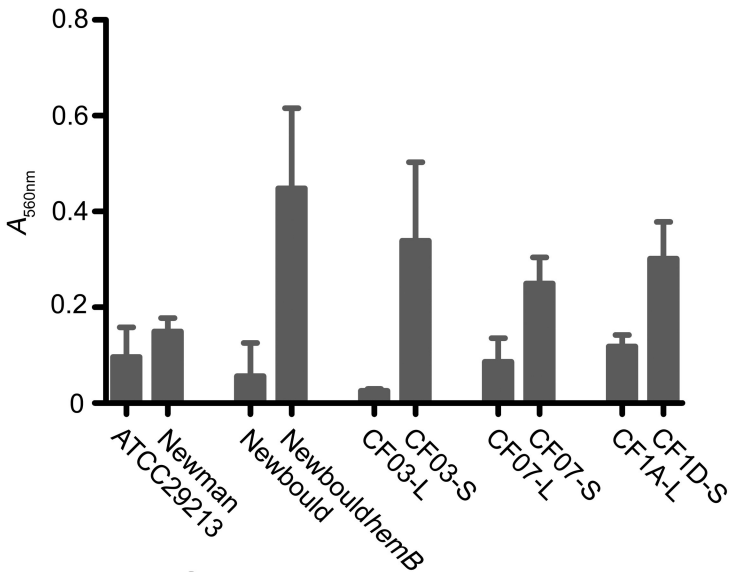

Additional file 6.

Supplement: Additional file 6 — Non-normalized absorbance values at 560 nm representing biofilm production for each of the strains used in Fig. 2. Non-normalized absorbance values at 560 nm representing biofilm production for each of the strains used in Fig. 2. Results show that strains vary in their relative production of biofilms but that for each related pairs of normal and SCV strains, SCV counterparts always produce more biofilm than their respective normal strains. [file 1471-2180-10-33-S6.PDF]
