# Supplementary material for: Staphylococcus aureus sigma B-dependent emergence of small-colony variants and biofilm production following exposure to Pseudomonas aeruginosa 4-hydroxy-2-heptylquinoline-N-oxide
Source: BMC Microbiol. 2010 Jan 30;10:33. doi: 10.1186/1471-2180-10-33 (PMC2824698; doi:10.1186/1471-2180-10-33)
Supplement: Additional file 2 — Minimal inhibitory concentrations (MICs) of gentamicin for the studied strains. Results of this file show that MICs of gentamicin for SCVs are of 8 μg/ml whereas those of normal strains are below 2 μg/ml. [file 1471-2180-10-33-S2.PDF]

Minimal inhibitory concentrations (MICs) of gentamicin for the studied strains

Strains	μg/ml
ATCC29213	0.5-1
Newman	1
Newbould	0.5-1
NewbouldΔ*sigB*	0.5-1
Newbould*hemB*	8
CF03-S	8
CF03-L	0.5-1
CF07-S	8
CF07-L	1
CF1D-S	8
CF1A-L	1

Additional file 2.
