# Supplementary material for: Staphylococcus aureus sigma B-dependent emergence of small-colony variants and biofilm production following exposure to Pseudomonas aeruginosa 4-hydroxy-2-heptylquinoline-N-oxide
Source: BMC Microbiol. 2010 Jan 30;10:33. doi: 10.1186/1471-2180-10-33 (PMC2824698; doi:10.1186/1471-2180-10-33)
Supplement: Additional file 4 — Auxotrophism found among HQNO-induced SCVs. Auxotrophism found among HQNO-induced SCVs generated from the normal cystic fibrosis strains CF07-L and CF1A-L. [file 1471-2180-10-33-S4.PDF]

Auxotrophism of SCVs recovered after an exposure to HQNO

Auxotrophism	Number of SCV colonies for each strain exposed to HQNO	
	CF07-L	CF1A-L
Hemin	0	0
Menadione	15	12
Thymidine	0	0
Unknown	1	1

Additional file 4.
